# Neuromorphic VLSI Models of Selective Attention: From Single Chip Vision Sensors to Multi-chip Systems

**DOI:** 10.3390/s8085352

**Published:** 2008-09-03

**Authors:** Giacomo Indiveri

**Affiliations:** Institute of Neuroinformatics, UZH-ETH Zurich, Winterthurerstrasse 190, 8052 Zurich, Switzerland

**Keywords:** Selective attention, winner-take-all (WTA), neuromorphic, Address-Event Representation (AER), integrate and fire (I&F) neuron

## Abstract

Biological organisms perform complex selective attention operations continuously and effortlessly. These operations allow them to quickly determine the motor actions to take in response to combinations of external stimuli and internal states, and to pay attention to subsets of sensory inputs suppressing non salient ones. Selective attention strategies are extremely effective in both natural and artificial systems which have to cope with large amounts of input data and have limited computational resources. One of the main computational primitives used to perform these selection operations is the *Winner-Take-All* (WTA) network. These types of networks are formed by arrays of coupled computational nodes that selectively amplify the strongest input signals, and suppress the weaker ones. Neuromorphic circuits are an optimal medium for constructing WTA networks and for implementing efficient hardware models of selective attention systems. In this paper we present an overview of selective attention systems based on neuromorphic WTA circuits ranging from single-chip vision sensors for selecting and tracking the position of salient features, to multi-chip systems implement saliency-map based models of selective attention.

## 1. Introduction

Processing detailed sensory information in real-time is a computationally demanding task for both natural and artificial sensory systems: if the amount of information provided by the sensors exceeds the parallel processing capabilities of the system, as is usually the case for example with vision systems, an effective strategy is to select *sub-regions* of the input and process them serially, shifting from one sub-region to another, in a sequential fashion [1, 2]. In biology this strategy is commonly referred to as *selective attention*. In primates selective attention plays a major role in determining where to center the high-resolution central foveal region of the retina [3, 4], by biasing the planning and production of saccadic eye movements [5, 6]. In artificial systems the same strategies can be used to decide which regions of the sensory input space to process, dramatically reducing the bandwidth requirements for information transfer, and the system's overall computational load.

In biology visual attention mechanisms have two main types of dynamics: a transient, rapid, bottom-up, task independent one [4], and a slower, sustained one, which acts under voluntary control [7]. Much of the applied research has focused on modeling the bottom-up aspect of selective attention. As a consequence, several software [8, 9] and hardware models [10–13] based on the concept of *saliency map, winner-takes-all* (WTA) competition, and *inhibition of return* (IOR) [14] have been proposed. Here we focus on hardware implementation of such selective attention systems on compact, low-power, hybrid analog/digital VLSI chips. Specifically, in the following sections we will show how it is possible to implement models of bottom-up selective attention mechanisms using WTA networks implemented in VLSI technology with neuromorphic circuits.

### 1.1. Neuromorphic Circuits

Neuromorphic circuits are a class of hybrid analog/digital electronic circuits inspired by the organizing principles of animal neural systems, implemented using standard Complementary Metal-Oxide Silicon (CMOS) VLSI technology, which explicitly implement biological-style processing on individual chips or systems composed of chips [15, 16]. These circuits are parallel and asynchronous, and they respond in real time. They operate in the sub-threshold regime (that is, with transistors that have gate-to-source voltage differences below their threshold voltage), where the transistors have physical properties that are useful for emulating neurons and neural systems, such as thresholding, exponentiation, and amplification [17].

Artificial sensory systems have already been implemented using conventional CMOS sensors interfaced to digital processing systems that execute computer algorithms on general-purpose serial or coarsely parallel architectures. However, these conventional digital systems tend to have excessive power consumption, size, and cost for useful real-time or robotic applications. This is especially true for conventional machine vision systems for which, with few exceptions, typical performance figures fall well short of robust real-world functionality.

Neuromorphic vision systems are based on custom unconventional sensory devices that process images directly at the focal plane level. These sensors typically use circuits which implement hardware models of the first stages of visual processing in biological systems [18, 19]. In the retina, early visual processing is performed by receptors and neurons arranged in a manner that preserves the retinal topography with local interconnections. Neuromorphic circuits have a similar physical organization: photoreceptors, memory elements, and computational nodes share the same physical space on the silicon surface and are combined into local circuits that process, in real-time, different types of spatio-temporal computations on the continuous analog brightness signal.

The highly distributed nature of physical computation in neuromorphic systems leads to efficient processing that would be computationally expensive on general-purpose digital machines. For example, like their biological counterparts, neuromorphic sensors such as VLSI silicon retina devices [20–22] can operate over an input range covering many orders of magnitude, despite limited bandwidth. This extraordinary performance is achieved by a simple but densely parallel process that involves continually adapting local reference signals to the average signal statistics prevailing there.

The similarities with biology, the dense processing, small size, and low power characteristics of neuromorphic VLSI circuits make them a convenient medium for constructing artificial sensory systems that implement saliency-based selective attention models.

### 1.2. Saliency-based models of selective attention

**Figure 1.**
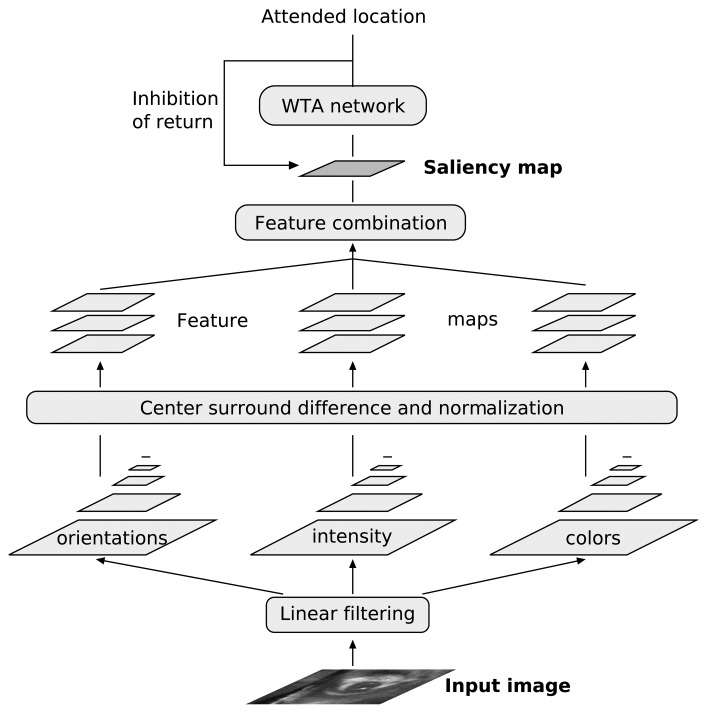
Schematic diagram of a saliency based model of selective attention.

In computer- and neuro-science several computational models of selective attention have been proposed [2, 6, 23–25]. Some of these models are based on the concept of “dynamic routing” [23], by which salient regions are selected by dynamic modification of network parameters (such as neural connection patterns) under both top-down and bottom-up influences. Other models, based on similar ideas, promote the concept of “selective tuning” [24]. In these models, attention optimizes the selection procedure by selectively tuning the properties of a top-down hierarchy of winner-take-all processes embedded within the visual processing pyramid. The types of models that we implemented in hardware are the bottom-up models based on the concept of the “saliency map”, originally put forth by Koch and Ullman [14]. These biologically plausible types of models account for many of the observed behaviors in neuro-physiological and psycho-physical experiments and have led to several software implementations applied to machine vision and robotic tasks [8, 9]. They are especially appealing to us because they lend themselves nicely to hardware implementations. A diagram describing the main processing stages of such type of model is shown in Fig. 1. A set of topographic feature maps is extracted from the visual input. All feature maps are normalized and combined into a master *saliency map*, which topographically codes for local saliency over the entire visual scene. Different spatial locations then compete for largest saliency, based on how much they stand out from their surroundings. A WTA circuit selects this most salient location as the focus of attention. The WTA circuit is endowed with internal dynamics, which generate the shifts in attention based on a mechanism named *inhibition of return* (IOR) (a key feature of many selective attention systems) [26].

As saliency-based selective attention models are massively parallel and highly modular, they lend themselves to VLSI implementation using analog neuromorphic circuits both on single-chip and multi-chip systems. In the following sections we will present examples of both kinds of systems, pointing out the advantages and disadvantages of both approaches.

## 2. Single-chip selective attention systems

Single-chip selective attention systems have been implemented mainly for modeling *visual* attention. Several neuromorphic attention systems of this kind have been proposed in the past [10–12, 27]. These systems typically contain photo-sensing elements and processing elements on the same focal plane, apply the competitive selection process to visual stimuli sensed and processed by the focal plane processor itself, and perform visual *tracking* operations.

These types of compact vision sensors are particularly useful in robotic applications and machine vision systems that carry out *active vision* tasks. Indeed tracking features of interest as they move in the environment is a computationally demanding task for machine vision systems in general. The control loop of active vision systems, comprising motors that steer the visual sensor, relies on the speed of the specific computation carried out. The stability of system depends on the latency of the sensory-motor control loop itself. Single-chip neuromorphic tracking sensors can dramatically reduce this latency and improve the performance of active vision systems.

Here we describe two single-chip selective attention systems for visual tracking that reduce the computational cost of the processing stages interfaced to them. These sensors carry out most of the computation on the focal plane itself and transmit only the final result of this computation. As they do not have to transmit vasts amounts of data that represent the raw input image to further processing stages, bandwidth and power requirements are greatly reduced. The tracking architectures described here differ from previously proposed ones in two key features: they select high-contrast edges independent of the absolute brightness of the scene (as opposed to simply selecting the scene's brightest region [10, 12, 27]); and use a hysteretic WTA network with positive feedback and lateral coupling, to lock-onto and smoothly track the selected targets (different from WTA networks used in other tracking devices [10, 12, 28, 29]).

The first chip implements a one-dimensional tracking architecture, while the second one implements a two-dimensional array.

## 3. A one-dimensional tracker chip

**Figure 2.**
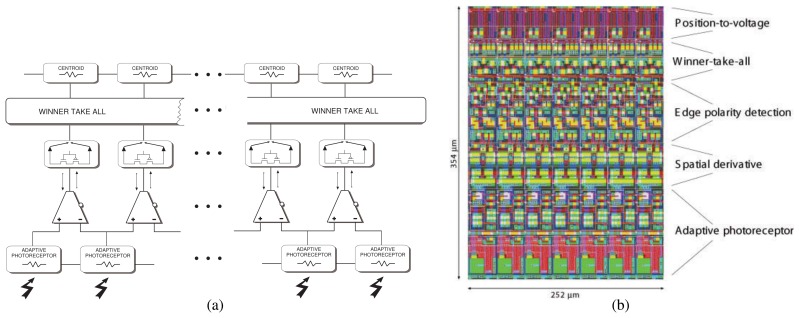
(a) Block diagram of single-chip tracking system. Spatial edges are detected at the first computational stages by adaptive photo-receptors connected to transconductance amplifiers. The edge with strongest contrast is selected by a winner-take-all network and its position is encoded with a single continuous analog voltage by a position-to-voltage circuit. (b) Portion of the chip layout containing 7 processing columns.

This device implements a 1D tracking architecture structured in a hierarchical way, comprising 5 processing stages (see Fig. 2(a)). The layout of the chip was designed by combining long and thin columns with the circuits implementing the 5 processing stages extending over the vertical dimension, in a way to optimize the area used and increase the number of pixels on the device (see Fig. 2(b)). The chip was fabricated using a standard 1.2*μ*m CMOS technology and occupies an area of approximately 2×2mm. The chip has a total of 40 processing columns, each 36*μ*m wide.

Image brightness data is processed in parallel through five main computational stages. A block diagram of the device's architecture is depicted in Fig. 2(a). The first stage is an array of adaptive photo-receptors that map logarithmically image intensity into their output voltages and which can be tuned to specific velocity ranges [30, 31]. The second stage is composed of an array of simple transconductance amplifiers, operated in the sub-threshold regime, which receive input voltages from neighboring pixels [17]. The amplitude of their output currents encode the contrast intensity of edges and the sign their polarity. At the third computational stage the polarity of each edge is gated so that the sensor selectively responds either to ON edges (dark to bright transitions), to OFF edges (bright to dark transitions), or to both. The fourth stage uses a hysteretic WTA network which selects and locks onto the feature with strongest spatial contrast moving at the speed that best matches the photoreceptor's velocity tuning. Finally in the last stage there is a position-to-voltage circuit [32], that allows the system to encode the spatial position of the WTA network's output with a single analog value. The layout of these circuits is shown in Fig. 2(b), and their functionality is described in the following paragraphs.

### 3.1. Adaptive photoreceptor

**Figure 3.**
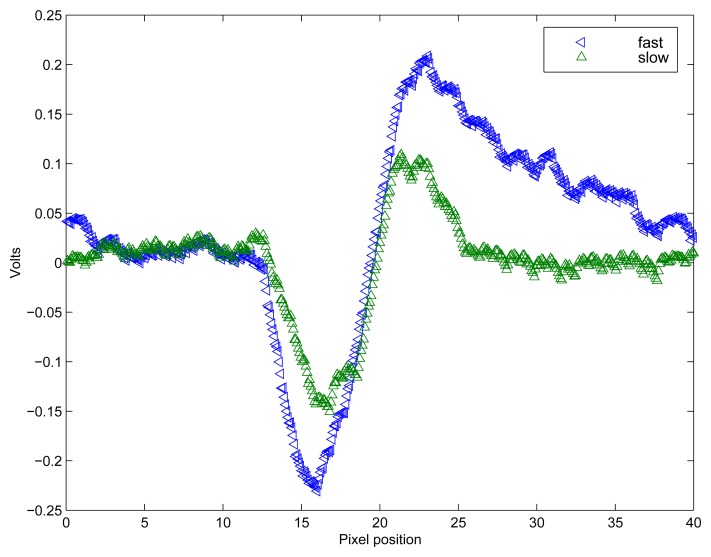
Response of the array of adaptive photo-receptors, to a dark bar on a white background moving from right to left with an on-chip speed of 31mm/s. Depending on the value of the adaptation bias setting, the photo-receptors can be tuned to respond to specific velocities. The trace with wider amplitude (left pointing triangles) has an adaptation tuning for fast speeds, while the trace with the smaller amplitude (upward pointing triangles) has a slightly slower speed tuning. The DC value of both responses was removed for clarity.

The adaptive logarithmic photoreceptor circuit is the one presented in [30], based on the design originally proposed in [31]. This circuit has been used extensively in many neuromorphic sensors. The response of the circuit is proportional to the image contrast and is largely invariant to absolute light intensity. The adaptive photoreceptor exhibits the characteristics of a temporal bandpass filter, with adjustable high and low frequency cut off values. Fig. 3 shows the response of the array of photo-receptors to a moving bar, for two different adaptation settings. Because of its adaptation property, this photoreceptor circuit has a response which results in both contrast and speed dependence.

### 3.2. Spatial derivative

Spatial derivative is implemented using simple transconductance amplifiers operated in the sub-threshold regime. The amplifiers receive input voltages from neighboring photo-receptors and provide a bidirectional output current that is proportional to the hyperbolic tangent of their differential input [17]. The output current saturates smoothly as the differential voltage increases (in absolute value) beyond 200 – 300mV. The possibility of electronically smoothing the input image (at the adaptive-photo-receptors stage) allows the user to operate the spatial derivative circuit always in its linear range, for a stimulus with fixed spatial frequencies. Furthermore, the presence of multiple stimuli with contrast high enough to saturate the transconductance amplifiers currents is not going to compromise the sensor's tracking performance, as the hysteretic WTA network is able to lock onto the feature selected.

### 3.3. Edge-polarity detection

The polarity of edges in the visual scene (light-to-dark or dark-to-light edges) is encoded by the sign of the transconductance amplifiers' currents. Each of these currents is fed into a circuit which rectifies the currents separating the positive component of the input current from the negative one [33]. The output current of the polarity selection circuit can therefore represent either OFF edges (light-to-dark), ON edges (dark-to-light) or both types of edges. The output currents of all edge-polarity detector circuits in the array are sourced in parallel to the elements of the next processing stage: the hysteretic WTA network.

### 3.4. Winner-take-all

This is the most important computational primitive for implementing models of selective attention mechanisms.

It has been argued that winner-take-all networks can implement faithful models of cortical processing and can account for many response properties of cortical networks [34–36]. The WTA circuit used in this work implements a simplified abstract model of these types of neural networks, and is based on the current-mode implementation originally proposed by Lazzaro *et al*. [37] almost 20 years ago. Its a fully analog circuit which still remains one of the most compact and elegant designs of analog current-mode WTA circuits: it is asynchronous; it responds in real-time; and it processes all its input currents in parallel using only two transistors per node, if the output signal is a voltage, or four transistors if the output signal is a current. We introduced some extensions to the basic design described in [37] that endow the WTA circuit with local excitatory feedback and with distributed hysteresis, via lateral coupling among WTA cells [38]. Local excitatory feedback enhances resolution and speed performance of the circuit, providing a hysteretic mechanism that withstands the selection of other potential winners unless they are stronger than the selected one by a set hysteretic current. Lateral coupling allows the winning input to shift between adjacent locations maintaining its winning status, without having to reset the network and imposes a smoothness constraint onto the focus of attention: cells adjacent to the winning pixel will hence be facilitated in the winner computation process whereas cells in the periphery will be inhibited.

**Figure 4.**
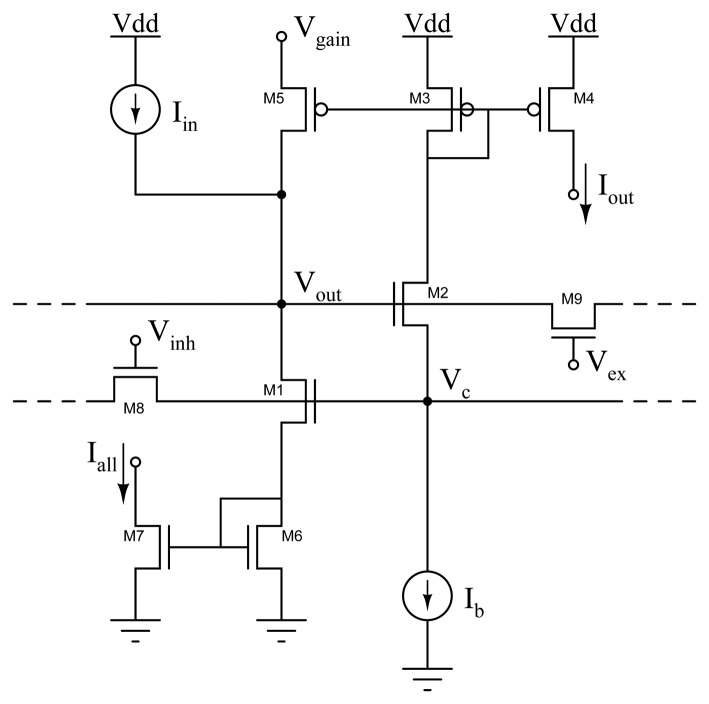
Hysteretic WTA circuit diagram, with local excitatory feedback, lateral excitatory coupling, lateral inhibitory coupling and diode-source degeneration.

The schematic diagram of this circuit is shown in Fig. 4. The current source that generates the bias current *I_b_* is implemented using a single n-type MOS transistor operated in the subthreshold domain [17]. In practical applications *I_b_* can be set by providing an external bias current into a single diode-connected transistor that has its gate connected to all the network's bias transistors (thus implementing a series of current-mirrors). Similarly, the input current source that generates *I_in_* can be implemented using a p-type transistor operating in the subthreshold regime. Although the WTA circuit can operate both in the weak and strong inversion regimes, it is typically operated in the weak inversion/subthreshold regime. In this regime the circuit is particularly sensitive to device mismatch and noise. In the existent implementation, when operated in subthreshold, the circuit selects one single winner if its input currents differ by at least 10% among each other, and one input is greater than the others.

Lateral excitatory connections allow the network to smooth input currents spatially, and distribute locally the hysteretic feedback current, while lateral inhibitory connections allow the network to select multiple winners, provided they are sufficiently distant from each other [38].

This enhanced type of WTA network is able to select and lock onto the input with strongest amplitude, and to track it as it shifts smoothly from one pixel to its neighbor [39]. This solution takes into account the assumption that the features being selected move continuously in space, and ensures that once the WTA network has selected a target and is engaged in visual tracking, it locks onto it and is not influenced by possible distracting stimuli in the periphery. Next to the cells in all the pixels, the hysteretic WTA network has an additional border cell with a constant input, set via an external voltage reference. This additional input is used to set a threshold for the spatio-temporal contrast of edges present in the scene: if the external input is stronger than all other inputs, then the border pixel of the WTA array wins, signaling the absence of high-contrast edges in the visual scene.

**Figure 5.**
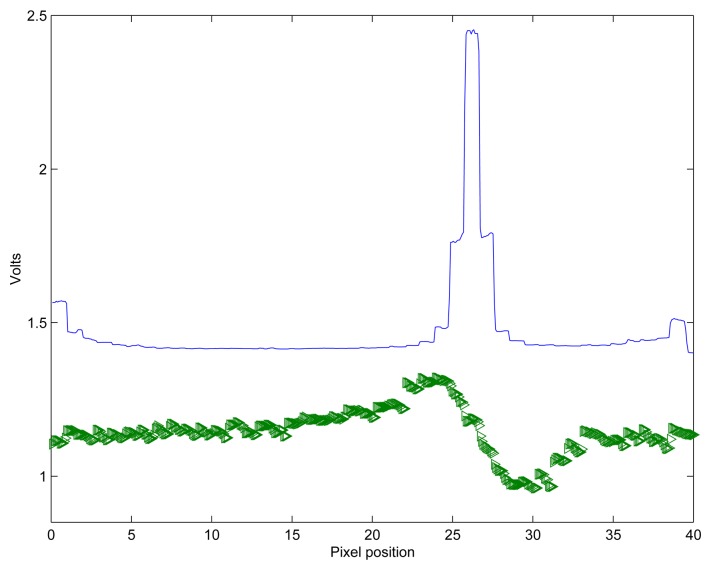
Response of the WTA network to the ON-edge of a bar moving from left to right at an on-chip speed of 31mm/s. The top trace represents the scanned WTA output of the 40 pixels, while the bottom trace represents the outputs of the adaptive photo-receptors.

Fig. 5 shows an example of the response of the WTA network to a moving high-contrast bar. The top trace of the figure represents the net input current to the WTA network, and shows the effect of spatial smoothing of the sum of input currents with the hysteretic current from the winner's positive feedback loop. It is clear from this figure that the active winning cell is the one corresponding to pixel 26. The bottom trace shows the instantaneous response of the adaptive photoreceptor array. The input stimulus was a 1cm-wide black bar on a white background positioned at approximately 17cm away from the focal plane and imaged onto the chip through a 4mm lens moving from left to right with an on chip speed of 31mm/s.

### 3.5. Position-to-voltage

This circuit, originally proposed in [32], comprises a parallel array of voltage followers with a common global output voltage, which receive inputs from nodes with increasing voltage references, distributed along a linear resistive network. The output currents generated by the WTA pixels at the previous stage, are used as bias currents for the followers. As only one WTA current is typically active at a time, the follower connected to the winning pixel will override all the others. The analog voltage on the winning node along the resistive network is buffered by the follower to the output pad. This single analog voltage therefore encodes the position of the winning pixel in the 1D array. Examples of P2V output traces are shown in Fig. 8, for the 2D tracker-chip case.

### 3.6. 1D tracker applications

**Figure 6.**
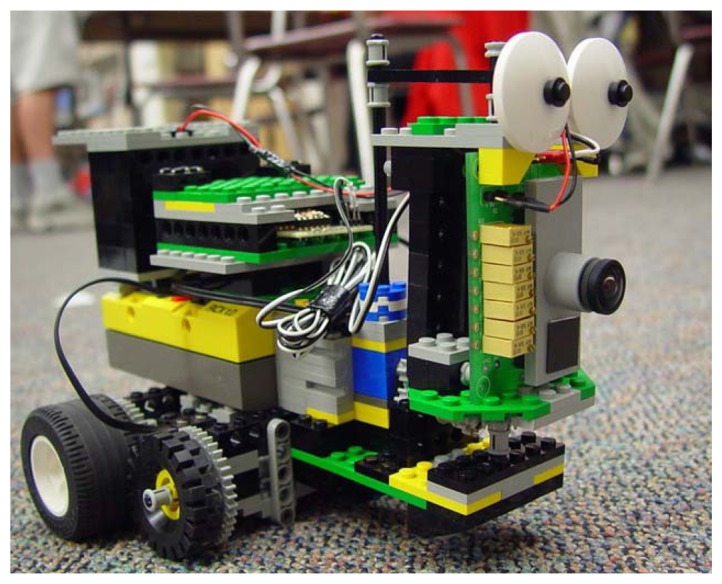
Tracker chip mounted on a LEGO robot performing a “target exploration task”. Using very little CPU power, this robot is able to simultaneously *explore* (make random body/head movements), *attend* (orient the sensor toward high-contrast moving edges) and *pursuit* (drive towards the target).

One dimensional tracking chips have been used in a wide variety of applications, especially in the field of mobile robotics [33, 40–42]. In this domain typical applications require compact and power-efficient computing devices which should be robust to noise, tolerant to adverse conditions induced by the motion of the system (*e.g*. to jitter and camera calibration problems) and possibly able to adapt to the highly variable properties of the world. To demonstrate these features, and to show how the tracking sensor can greatly reduce the computational load of the system's CPU we interfaced the 1D tracking sensor to a LEGO robot, controlled by a very inexpensive Mindstorms RCX micro-controller (see Fig. 6). Using simple control algorithms the roving robot was able to reliably track lines randomly layed out on the floor, for a wide variety of conditions (*e.g*. floors with different texture, cables of different colors and sizes, extreme illumination conditions, etc.), and to implement basic navigation behaviors [43].

Analogous experiments were also carried out using larger robotic platforms, such as the *Koala* mobile robot (K-Team, Lausanne), and characterizing more thoroughly the robot's tracking performance [33].

Given that all the circuits implemented in the 1D tracking chip operate in a massively parallel fashion, and that the processing time does not scale with array size, it is possible to extend the design to 2D architectures, with minimal effort.

## 4. A two-dimensional tracker chip

**Figure 7.**
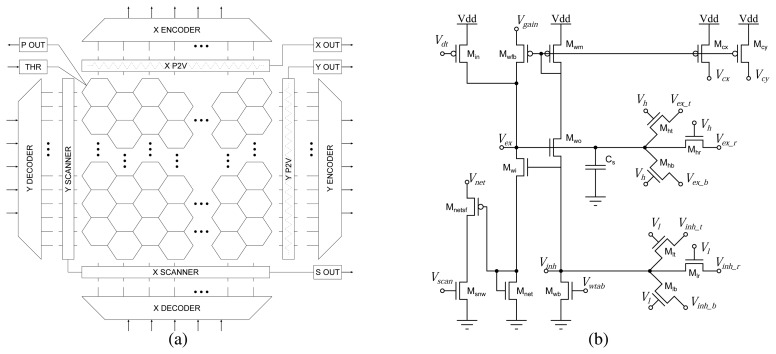
(a) The two-dimensional tracker chip architecture. (b) Circuit diagram of a two-dimensional hysteretic WTA network.

The 2D tracking chip comprises a core array of 26 × 26 pixels arranged on a hexagonal grid, with peripheral analog and digital circuits for input/output (I/O) operations (see Fig. 7(a)). The chip was implemented using a standard 0.8 *μ*m CMOS process and occupies an area of merely 3.22 mm×2.56 mm. To reduce the layout area of the individual pixels in the array we used a reduced set of circuits, compared to those used in the 1D architecture columns. Each pixel comprises a photo-sensing stage, a hysteretic WTA circuit with spatial coupling, and interfacing I/O circuits. The photo-sensing stage used in this sensor differs slightly from the one used in the 1D sensors, in that the photoreceptor circuits respond to contrast *transients* (rather then to absolute contrast). This property makes sensor sensitive to temporal changes in the scene contrast, rather than spatial edges, as for the 1D case. The transient differentiating photo-receptor circuit has been described and fully characterized in [44]. Large changes in luminance activate a transient current in the circuit, which is fed into the 2D hysteretic WTA network. At the output stage, the chip comprises both analog P2V circuits, and additional *digital* output circuits, to encode the position of the winner. The chip has also on-chip scanners and address decoders. The former are used to read the DC response of the adaptive photoreceptor array (*e.g*. for displaying images on monitors); while the latter are used to access each pixel in a random-access mode (*e.g*. for reading out sub-regions of the image). A particularly interesting feature is given by the fact that the input address decoders can be directly connected to the chip's digital outputs (which encode the position of the winning pixel) for selectively reading the photoreceptor output of just that pixel, and displaying only the part of the image that is of interest. Larger regions of interest can be selectively accessed by addressing small windows around the winning pixel's address (*e.g*. with the aid of a micro-controller).

### 4.1. The 2-D hysteretic winner-take-all circuit

The basic cell of the 2D hysteretic WTA network shown in Fig. 7(b). It is the 2D extension of the circuit used in the 1D tracking chip. The output current of the photoreceptor stage is copied into the node *V_ex_*. If the input current to the considered pixel is the strongest, the cell “wins” and transistors M_cx_ and M_cy_ source an output current proportional to the circuit's bias current, set by *V_wtab_*, bringing the output voltages *V_cx_* and *V_cy_* high. Voltages *V_cx_* of all pixels belonging to common columns are tied together, and voltages *V_cy_* of all pixels belonging to a common row are tied together. A copy of the WTA bias current, attenuated exponentially by the bias voltage *V_gain_* is fed back into the input node, via M_wfb_. Transistors M_ht_, M_hb_, and M_hr_ diffuse the currents coming from M_in_ and M_wfb_ to the *V_ex_* nodes of the three (top, bottom, and right) neighboring cells. The bias voltage *V_h_* is used to tune the diffusion space constant and to control the amount of lateral excitatory coupling. Conversely, transistors M_lt_, M_lb_, and M_lr_ implement the inhibitory coupling among neighboring cells. The bias voltage *V_l_* is used to control the spatial extent of lateral inhibition. If *V_l_* is set to *V_dd_*, inhibition is global, and only one pixel in the whole array can win.

The current flowing through M_net_ represents the net current that the WTA cell is receiving, corresponding to sum of the input current from the photoreceptor circuit, the positive-feedback current and the diffused excitatory currents. The voltage *V_net_*, logarithmically proportional to this net current, can be scanned out to image the overall network activity and view the relative effects of positive feedback current modulation(*V_gain_*), and excitatory and inhibitory coupling modulations (*V_h_* and *V_l_* respectively).

### 4.2. Peripheral I/O circuits

The peripheral output circuits comprise both analog position-to-voltage (P2V) circuits, and digital position encoding circuits, for reading out the output of the WTA network [45]; in addition an analog source-follower circuit is used to read-out the state of selected individual pixels, and a 2D scanner [46] can be used for displaying the state of all pixels on an external monitor.

The peripheral input circuits comprise input address decoders to select the pixel to monitor with the source-follower.

**Figure 8.**
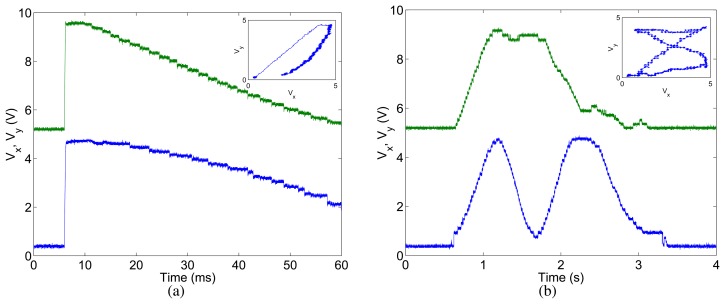
(a) Output of the analog P2V circuits in response to a target moving from the right top corner to the bottom central part of the sensor's field of view. The bottom trace (*V_x_*) reports the *x* position of the target. The top trace (*V_y_*), offset in the plot by 5V for sake of clarity, reports the *y* position of the target. The inset shows *V_y_* versus *V_x_*. (b) Output of the analog P2V circuits in response to a target moving from the bottom left corner to the top right one, on to the top left, to the bottom right, and back to the bottom left corner. (b) Circuit diagram of a two-dimensional hysteretic WTA network.

In Fig. 8(a) we show experimental results obtained by enabling the analog P2V circuits and measuring their output voltages *V_x_* and*V_y_* which encode the *x* and *y* position of the winning pixel. The WTA network was biased in a way to have local excitation (*V_h_* of Fig. 7(b) was set to 0.8V) and global inhibition (*V_l_* was set to *V_dd_*). The measurement shows the sensor's response to a target appearing in the upper right corner of the sensor's field of view and quickly moving downward and to the right. Before the target appeared, the sensor's output was sitting around *V_x_* ≈ 0V and *V_y_* ≈ 0V. This is because the bottom-left pixel (0,0) receives an additional input current, set by an external bias voltage *V_thr_*, that sets a global threshold: if no visual stimulus is strong enough to overcome this threshold, the output is always “zero”. As soon as the target appeared in the sensor's field of view, the WTA network switched winner, and the P2V circuits modified *V_x_* and V*y* accordingly. The response time of the WTA and P2V circuits combined, at the onset of the stimulation, is about 200*μ*s. The switching time, required to report a change of winner from one pixel to its nearest neighbor, is around 15*μ*s.

In Fig. 8(b) we show the response of the sensor to a target appearing in the bottom left corner of the field of view, slowly moving to the top right corner and then completing a figure-eight pattern. Note the different time scales in Figs. 8(a) and 8(b).

In both experiments the target was the light spot of a laser-pointer shone on a flat surface 30cm from the chip's focal plane. Images were focused onto the focal plane using an 8mm lens with an *f* –number of 1.2. The sensor's response does not depend on the background onto which the target is overlaid, nor does it change with absolute background illumination.

**Figure 9.**
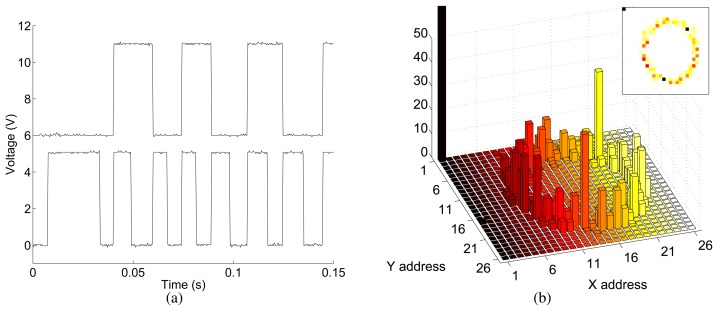
(a) Output of least significant bit (bottom trace) and second-least significant bit (top trace, displaced by 6V) of the ‘X’ address in response to a target moving from right to left. (b) Histogram of the addresses measured from the sensor's address encoders in response to a target moving on a circular trajectory.

By switching the state of the demultiplexer connected to the WTA outputs we disabled the analog P2V circuits and enabled the asynchronous address encoders. Figure 9(a) shows the the response of two address lines (the least significant and second-least significant bits of the X address) in response to the same stimulus of Fig. 8(b) moving from right to left. The non-uniform pulse widths are due to the asynchronous response of the circuit to the variable speed of the stimulus. In a second experiment, we placed the sensor in front of a CRT monitor, showed a white box performing a circular motion on a black background, and sampled the chip's address encoder outputs every 25ms over a period of 40s. In this period the target made 16 full revolutions. The histogram of the sampled addresses is shown in Fig. 9(b). As the global threshold was set relatively high, address (1,1) was selected most often (193 samples, off-scale in the figure).

The response time of the sensor to the sudden appearance of a target is 1.2*μ*s when the digital outputs are enabled, and can be as long as 6*μ*s when the analog outputs are enabled. Power consumption is also dependent on the output mode selected: with digital outputs (and no scanners) enabled, the system dissipates 1.1mW, while in the analog output mode the system dissipates 600*μ*W.

This device represents a compact, low-power, single-chip selective attention system in which images are sensed and processed fully in parallel. The pixel reporting the strongest positive luminance transient (*e.g*. induced by a high-contrast moving target) is selected by the WTA network. Its position can be read out using either analog P2V circuits or digital address encoders. The sustained response of each photoreceptor and net input current to each WTA can be read out serially, using on-chip scanners, and displayed on monitors. Additionally, photoreceptor voltages can be individually sensed, using input address decoders. The WTA analog outputs can be used to drive motors and actuators, for example on small autonomous robots. The WTA digital outputs can be used to drive the input address decoders and read the photoreceptor output of only the winning pixel. This mechanism could be exploited (*e.g*. using a micro-controller) to selectively read out just the regions of the image around the position of the target, rather than reading out all the raw image data.

This and the previous single-chip system have great advantages, such as size, fabrication cost and low power consumption, and extraordinary computational capabilities. However, to design systems with greater computational power and higher flexibility one needs to resort to *multi-chip* systems.

## 5. Multi-chip selective attention systems

Neuromorphic multi-chip systems generally consist of systems containing one or more *sensory* devices, such as silicon retinas[21, 22], silicon cochleas [47] or vision sensors[48], interfaced to one or more chips containing networks of spiking neuron circuits. These chips can process the sensory signals (*e.g*. detecting salient regions of the sensory space [49], learning correlations [50], *etc*.) and eventually transmit the processed signals to actuators, thus implementing complete neuromorphic sensory-motor systems. Specifically, using multi-chip systems it is possible to implement more elaborate models of selective attention compared to what has been done on single-chip systems. Unlike for single-chip devices, multi-chip systems can decouple the sensing stage from the selective attention/competition stage. Therefore input signals need not arrive only from visual sensors, but could represent a wide variety of sensory stimuli obtained from different sources. In multi-chip selective attention systems multiple instances of the same selective attention chip could be used to implement feature normalization and combination stages in hierarchical selective attention architectures (see Fig. 1), sensors could be distributed across different peripheral regions of the system setup, and the input visual sensors could be high-resolution silicon retinas which do not have the small fill factors that single-chip 2D attention systems are troubled with. Furthermore the signals encoding the bottom-up generated saliency map sent to a selective attention chip can be merged with top-down modulating signals (*e.g*. from associative memory modules) to bias the competition process.

**Figure 10.**
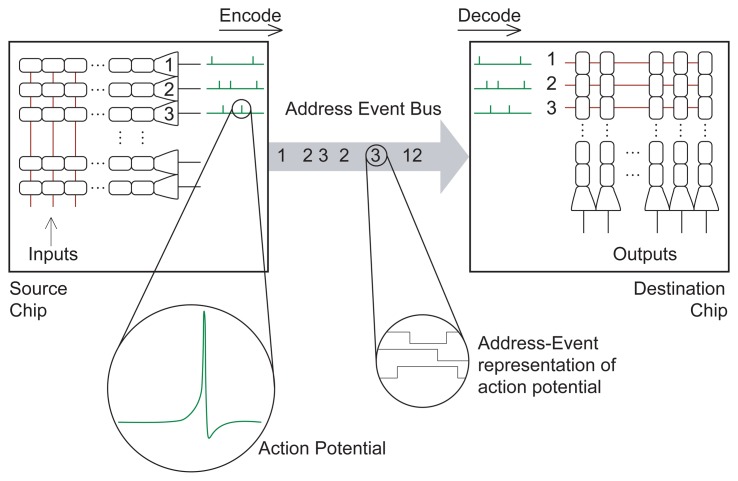
Schematic diagram of an AER chip to chip communication example. As soon as a sending node on the source chip generates an event, its address is written on the Address-Event Bus. The destination chip decodes the address-events as they arrive and routes them to the corresponding receiving nodes.

In these types of multi-chip systems analog signals are transmitted across chips using an asynchronous communication protocol based on the *Address-Event Representation* (AER, see Fig. 10) [51, 52]. In this representation analog signals are converted into streams of stereotyped non-clocked digital pulses (spikes) and encoded using pulse-frequency modulation (spike rates). When a spiking element on a VLSI device generates a pulse its address is encoded and instantaneously put on a digital bus, using asynchronous logic (see Fig. 10). In this asynchronous representation time represents itself, and analog signals are encoded by the inter-spike intervals between the addresses of their sending nodes.

By converting analog signals into a digital representation, we can take advantage of the considerable understanding and development of high-speed digital communications, emulating the parallel, but slow, connectivity of neurons using axons with fast, but serial, connectivity through digital buses. We basically trade-off “space” (the number of pins and wires that would be required to transmit spikes from each individual neuron on a chip) with “time”, exploiting the fact that our neuromorphic circuits have typical time constants of the order of milliseconds and digital buses have bandwidths of the order of MHz.

An important consequence of using a digital chip-interconnect scheme is the relative ease with which these chips are able to interface to existing digital hardware. From the simulation of input spike trains to quickly re-configuring a network's connectivity via address routers, the flexibility of software can be used to produce a more powerful modeling tool. From the engineering perspective, the translation of our analog signals into a stream of asynchronous spikes not only facilitates communication, it opens up new possibilities for the efficient implementation of both computation and memory in the spike domain.

In the case of single-sender/single-receiver communication, a simple handshaking mechanism ensures that all events generated at the sender side arrive at the receiver. The address of the sending element is conveyed as a parallel word of sufficient length, while the handshaking control signals require only two lines. Systems containing more than two AER chips (*e.g*. with AER sensors at the input stages, AER networks on neurons for doing the computation and AER read-out modules to drive possible actuators) are constructed by implementing special purpose off-chip arbitration schemes [53, 54].

### 5.1. AER selective attention chips

In addition to the single-chip tracker chips described in the previous Section, several additional VLSI chips that implement *visual* selective attention mechanisms have been presented [10–12]. These systems contain photo-sensing elements and processing elements on the same focal plane, and typically apply the competitive selection process to visual stimuli sensed and processed by the focal plane processor itself. Unlike these systems, the types of selective attention devices described in this Section are able to receive input signals from any type of AER device. Input signals need not arrive only from visual sensors, but could represent a wide variety of sensory stimuli obtained from different sources. The AER selective attention chips are able not only to *receive* AER signals, but also to *transmit* the result of its computation using the Address-Event Representation. With both input and output AER interfacing circuits the chip can be thought of as a VLSI “cortical” module able to receive and transmit spike trains.

**Figure 11.**
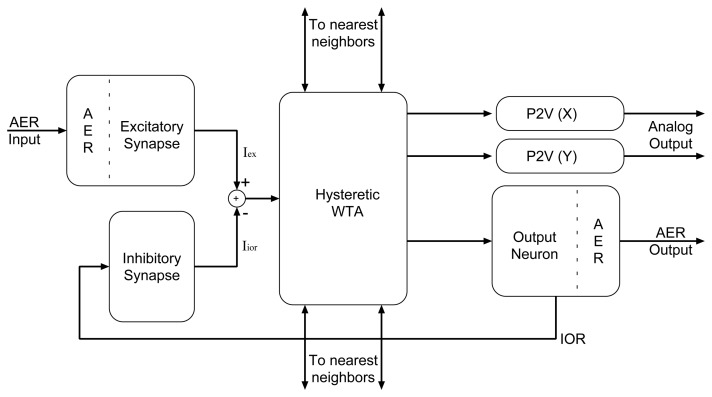
Block diagram of an AER selective attention chip pixel.

We implemented 2 generations of AER selective attention chips. The first one, comprising an array of 8 × 8 processing elements (pixels), has been fully characterized and described in [13]. The second one comprises an array of 32 × 32 pixels, implements several improvements over the previous implementation, and has been described in [55]. The specific pixel architecture in both AER selective attention chips is the same, and described in Fig. 11. In particular each pixel comprises an excitatory silicon synapse, an inhibitory silicon synapse, a hysteretic WTA circuit [39], an output integrate and fire (I&F) spiking neuron, and two P2V circuits.

The input excitatory synapses receive spike trains from external devices and provide an excitatory current to the local WTA circuit. The WTA cells compete among each other until the one receiving the strongest net excitatory input wins the competition and inhibits all other cells. The winning WTA cell injects current into its corresponding I&F neuron, which produces spike trains at a rate proportional to its input current. The I&F neuron projects its spikes both to AER interfacing circuits, for transmitting the result of the computation off-chip to further processing stages, and to the pixel's local on-chip inhibitory synapse. The resulting inhibitory current is subtracted from its corresponding input excitatory current. This negative feedback loop implements the so called *inhibition of return* (IOR) mechanism [26, 56]: the spikes produced by the winning pixel are integrated by the inhibitory synapse, and as the inhibitory current increases in amplitude, the effect of the input excitatory current is diminished and eventually the WTA network switches stable state, selecting a different pixel as the winner. Note how the integrate and fire neurons, necessary for the Address-Event I/O interface, allowed us to implement the IOR mechanism by simply including an additional inhibitory synaptic circuit.

Depending on the dynamics of the IOR mechanism, the WTA network will continuously switch the selection of the winner between the strongest input and the second-strongest, or between the strongest and more inputs of successively decreasing strength, thus generating focus of attention *scan-paths*, analogous to eye movement scan-paths [57]. The dynamics of the IOR mechanism depend on the time constants of the excitatory and inhibitory synapses, on their relative synaptic strengths, on the input stimuli and on the frequency of the output inhibitory neuron.

**Figure 12.**
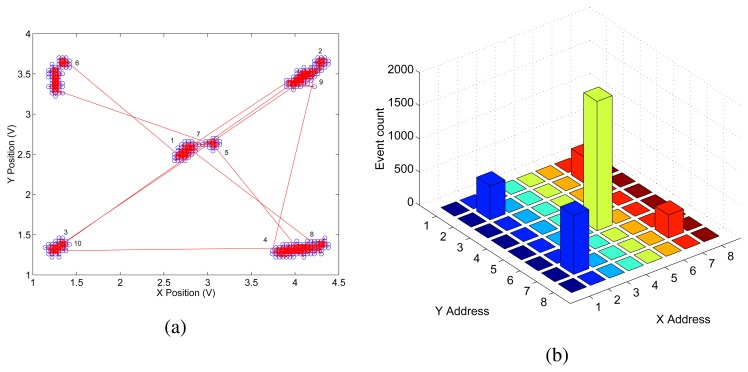
(a) Output of the P2V circuits of the selective attention architecture measured over a period of 300ms, in response to a test stimulus exciting four corners of the input array at a rate of 30Hz and a central cell at a rate of 50Hz; (b) Histogram of the chip's output address-events, captured over a period of 13.42s in response to the same input stimulus.

To characterize the behavior of the selective attention chips with well controlled input signals we first interfaced the to a workstation and stimulated them using synthetic AER spike trains. Figure 12 shows the result of an experiment with the 8 × 8 pixel selective attention chip: we used a test stimulus that excited cells (2,2) (2,7) (7,2) and (7,7) of the selective attention chip with 30Hz pulses, and cell (5,5) with 50Hz pulses. Figure 12(a) shows the analog output of the P2V circuits in response to 300ms of stimulation with this artificial “saliency map” input. As expected, the system initially selects the central cell (5,5). But, as the IOR mechanism forces the WTA network to switch the selection of the winner, the system cycles through all other excited cells as well. The P2V circuits are actively driven when the WTA network is selecting a winner. At the times in which no cell is winning (*i.e*. when all cells are inhibited), there is no active device driving the P2V circuits, and their outputs tend to drift toward zero. This is evident in Fig. 12(a), for example, at the position corresponding to cell (7,2) in the lower right corner of the figure. When the network selects it as its eighth target, the horizontal P2V circuit outputs approximately 4.4V and the vertical one outputs approximately 1.3V. When the IOR mechanism forces the network to de-select the winner the outputs of the P2V circuits slowly drift toward zero. As soon as inhibition decreases, the network selects the cell (7,7) as the new (ninth) winner, the position to voltage circuits are actively driven again, and their output quickly changes from approximately 3.6V and 1.2V to 4.2V and 3.5V (for the horizontal and vertical circuits respectively).

In Fig. 12(b) we plot the histogram of the chip's output address-events, captured over a period of 13.42s in response to the same input stimulus. As shown, the chip's AER output reflects, on average, the input stimulus and is consistent with the analog output measured with the P2V circuits.

### 5.2. Computer-generated saliency maps

To test the chips with more realistic saliency maps we used standard benchmark images used in the literature, and generated the saliency maps in software with the Matlab SaliencyToolbox [58].

Specifically, we computed saliency maps from *color, intensity* and *orientation* (at 0,45,90,135 degrees) feature maps. We then transformed the saliency maps into appropriate AER spike-train signals as inputs to the AER selective-attention chips: for each pixel, we generated a spike train with a frequency proportional to the pixel's saliency value.

**Figure 13.**
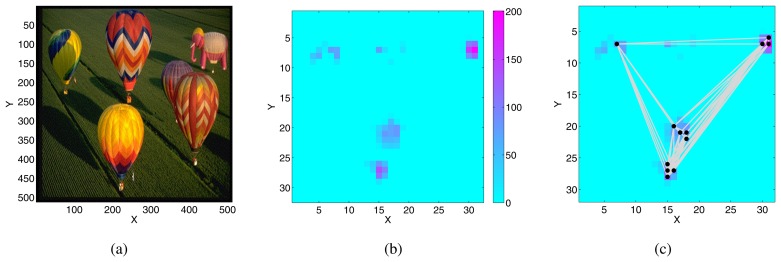
Focus of attention scan paths: (a) Input image used to generate the saliency map. (b) Saliency map obtained from the SaliencyToolbox, with the default parameters. The colors code for the saliency of the corresponding pixel in the input image. The intensities were re-scaled to the range 0 to 200, to represent the mean frequencies of the spike trains used to stimulate the chip. (c) Focus of attention scan path generated by the 32 × 32 selective-attention chip, superimposed on the saliency map. The black dots show the fixation points, the grey lines connect consecutive fixations.

Figure 13 shows an example input image, the software generated saliency-map, and the response of the chip to the corresponding input AER spikes.

By adjusting the parameters of the WTA, synapse and neuron circuits in the selective-attention chip, it is possible to produce focus of attention scan paths that match very closely the ones generated by the detailed computational models in the SaliencyToolbox [55]. The equivalence between computational model and VLSI behavior can be obtained for both spatial locations selection on the saliency map, and the dynamics of the IOR mechanism [55]. This demonstrates that the selective-attention chips represent a faithful implementation of bottom-up saliency-based selective attention models [4], and can be used as a real-time tool for both practical applications and for basic research in investigations of selective attention mechanisms.

### 5.3. AER sensor-generated saliency maps

To verify that these chips can be useful tools for practical applications, we interfaced them to AER silicon retinas, which produce a saliency map based on local changes in contrast, and implemented a stand-alone real-time selective attention multi-chip system.

The AER silicon retina sensor used in these experiments is the 64×64 pixels sensor designed by P. Lichtsteiner and T. Delbruck at the Institute of Neuroinformatics, and described in [59]. It generates asynchronous events corresponding to temporal changes in the logarithm of local image intensity. As $\frac{d}{dt}\log I=\frac{dI/dt}{I},$, where *I* is the pixel illumination, the retina output encodes temporal changes in contrast rather than absolute illumination differences. This property allows the retina to adapt to the global illumination level, responding to 20% contrast over a dynamic range spanning over 5 decades. Each pixel of the retina responds to both positive and negative variations in contrast, transmitted as ON and OFF events respectively. In this specific multi-chip system, both ON and OFF events are sent to selective attention chip, disregarding the polarity information. The 64 × 2 (ON and OFF) events of the retina are mapped onto the 32 × 32 pixels of the selective attention chip using a topographic 4(×2) : 1 linear mapping. As these address events are sent to the selective attention chip without any additional preprocessing, they represent a saliency map constructed using only contrast changes as relevant features. Using this “contrast transients” saliency map the chip determines where the focus of attention has to be deployed.

**Figure 14.**
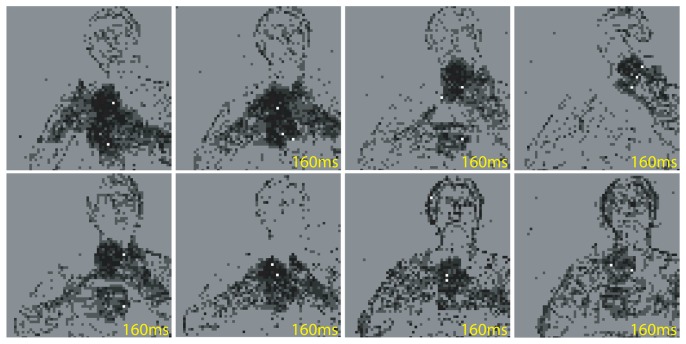
Selective attention with natural moving stimuli (moving hands of a person): screen shots of the recorded activity from the retina (black and grey pixels) and selective attention chip (white pixels). The numbers on the bottom right of each screen shot correspond approximately to the time gap from the previous screen shot. The hands are highly salient objects as they produce a strong local response in the retina. The moving arms and shoulders of the person are not as salient because the activity they elicit is more distributed. The edges of the head are selected even more rarely (second last screen shot).

In Fig. 14 we show the results of an experiment made using this multi-chip system with natural stimuli freely moving in front of the retina. Specifically, a person facing the retina was moving head, shoulders, and hands, while the activity of both AER chips was being monitored on a workstation. Figure 14 shows selected screen shots made by integrating all of the events read from both the retina (black and grey pixels) and selective attention chip (white pixels) over frames of 80ms, and displaying them in single images. The system often alternates between the two hands, rarely selecting the arms and shoulders. This behavior is obtained by appropriately setting the WTA later excitation settings, to give circumscribed regions of activity a competitive advantage with respect to single edges or isolated pixels. These settings are therefore useful for selecting and tracking multiple high-contrast moving objects, and shifting from one to the other with the IOR dynamics.

In the context of complex dynamic scenes, where the sensor itself moves and all the input changes at once, the role of IOR is less clear. Indeed, in experiments in which the silicon retina was mounted on a pan-tilt system, and was allowed to carry out saccadic-like movements, the system performed active tracking following the most salient moving target, without using IOR dynamics (see [55] for a detailed analysis).

As with the 1D and 2D tracking chips, also this multi-chip system can be used to selectively track high-contrast moving targets. The single chip solutions are more compact and dissipate much less power. However the multi-chip system offers a much larger degree of flexibility, and has the potential of allowing the construction to much more elaborate multi-modality selective attention systems. For example, it would allow to integrate multiple AER sensors at the input stage, use multiple instances of the same selective attention chip at an intermediate feature-extraction stage, and project all the feature map outputs to a top selective attention chip. In this last scenario, the parallel selective attention chips in the inter - mediate stage would carry out selective attention on the distributed feature/saliency maps, and the top selective attention chip would merge the multiple feature maps into a global saliency-map, and perform the top-level WTA and IOR operations, very much like it is done in elaborate software models [4] based on the architecture of Fig. 1.

## 6. Conclusions

We presented an overview of neuromorphic VLSI models of selective attention systems applied to visual tracking applications. Specifically we described examples of single-chip and multi-chip selective attention systems that make use of the technology developed over the last few years within the field of neuromorphic engineering. These examples are representative of what can be achieved using the present state-of-the-art. Up to now neuromorphic engineers have mastered the art of building single-chip systems. We are starting to consolidate the framework for designing and successfully implementing multi-chip AER systems, and mixed systems containing neuromorphic VLSI devices and conventional analog/digital electronics. We have reached the point where the technology is standardized and mature enough for building complex systems, containing sensory devices interfaced to chips carrying out different types of computation, interfaced to actuators interacting in real time with the environment [60]. The possibility to build complex neuromorphic systems which sense and interact with the environment will hopefully contribute to advancements both in basic research and in commercial applications. This technology is likely to become instrumental both for research on computational neuroscience, and for practical applications that involve sensory signal processing, in uncontrolled environments.
